# Identification of Diagnostic Biomarkers and Subtypes of Liver Hepatocellular Carcinoma by Multi-Omics Data Analysis

**DOI:** 10.3390/genes11091051

**Published:** 2020-09-06

**Authors:** Xiao Ouyang, Qingju Fan, Guang Ling, Yu Shi, Fuyan Hu

**Affiliations:** Department of Statistics, School of Science, Wuhan University of Technology, 122 Luoshi Road, Wuhan 430070, China; ouyangxiao@whut.edu.cn (X.O.); fanqingju@hotmail.com (Q.F.); ling_guang0@163.com (G.L.); shiyu87@whut.edu.cn (Y.S.)

**Keywords:** ensemble of decision trees, diagnostic biomarkers, LIHC subtyping

## Abstract

As liver hepatocellular carcinoma (LIHC) has high morbidity and mortality rates, improving the clinical diagnosis and treatment of LIHC is an important issue. The advent of the era of precision medicine provides us with new opportunities to cure cancers, including the accumulation of multi-omics data of cancers. Here, we proposed an integration method that involved the Fisher ratio, Spearman correlation coefficient, classified information index, and an ensemble of decision trees (DTs) for biomarker identification based on an unbalanced dataset of LIHC. Then, we obtained 34 differentially expressed genes (DEGs). The ability of the 34 DEGs to discriminate tumor samples from normal samples was evaluated by classification, and a high area under the curve (AUC) was achieved in our studied dataset and in two external validation datasets (AUC = 0.997, 0.973, and 0.949, respectively). Additionally, we also found three subtypes of LIHC, and revealed different biological mechanisms behind the three subtypes. Mutation enrichment analysis showed that subtype 3 had many enriched mutations, including tumor protein p53 (TP53) mutations. Overall, our study suggested that the 34 DEGs could serve as diagnostic biomarkers, and the three subtypes could help with precise treatment for LIHC.

## 1. Introduction

Liver hepatocellular carcinoma (LIHC), the primary malignancy of liver, is derived from hepatocytes and accounts for over 80% of cases of liver cancer. LIHC is predicted to be the sixth most commonly diagnosed cancer and the fourth leading cause of cancer deaths worldwide [1]. LIHC is too often found at the advanced disease stage, and at this stage, there is virtually no effective treatment that can improve survival rates [2]. Therefore, finding diagnostic biomarkers is essential for the early diagnosis and individualized treatments for LIHC. In addition, the correct discrimination of the subtypes is helpful to provide personalized therapy. 

Biomarkers have many potential applications in oncology, including screening, differential diagnosis, determination of prognosis, and monitoring the progression of disease [3]. Diagnostic biomarkers are used to improve the diagnosis of a disease. Diagnostic biomarker discovery, i.e., identifying important features that can discriminate tumors from normal samples, is commonly solved by different feature selection methods [4]. Many studies focused on identifying diagnostic biomarkers by finding differentially expressed genes (DEGs), as DEGs are the most informative genes among a large number of irrelevant ones. For example, Yin et al. used the integration of DEG screening and the method of weighted gene co-expression network analysis to identify biomarkers for hepatocellular carcinoma (HCC) [5]. Li et al. identified 273 DEGs as candidate targets for the diagnosis of HCC using GEO2R (http://www.ncbi.nlm.nih.gov/geo/geo2r) [6]. Kaur et al. identified a three-gene HCC biomarker based on DEGs [7].

However, most of these studies did not consider the imbalance problems between tumors and normal samples, which are likely to produce unreliable biomarkers. Typically, approaches to specifically deal with the imbalance problem are proposed from the data and algorithmic levels. Sampling methods consist of balancing the original dataset, either by under-sampling for the majority class or over-sampling the minority class [8,9]. Algorithm level data balancing is widely used, such as cost-sensitive learning [10,11], integration methods, and single class learning. More notably, other methods were also proposed, such as two-stage feature selection [12], which provided a new way of thinking about dealing with unbalanced data.

Molecular subtyping refers to finding clusters of tumors that have shared characteristics, which is helpful to the treatment of specific cancers. Molecular subtyping could help researchers to identify both actionable targets for drug design as well as biomarkers for response prediction [13]. Typically, cancers were classified using pathological criteria that rely heavily on the tissue site of origin [14]. However, new data-driven approaches have been proposed in cancer subtyping based on gene expression profiling. For example, non-negative factorization clustering with a standard “Brunet” method was conducted, and two distinct molecular HCC subtypes were identified [15]; statistical analysis was applied to HCC tumor examples to judge whether they can be divided into specific clusters with distinct features [16]. Therefore, data-driving methods might be useful to deal with problems in biology.

In this study, we first identified 34 DEGs of LIHC by an integration method which involved the Fisher ratio, Spearman correlation coefficient, classified information index, and the ensemble of decision trees (DTs). Then, we discussed two applications of the 34 DEGs: On the one hand, they were potential diagnostic biomarkers that showed good performance in discriminating tumors from normal samples; on the other hand, they were used to classify tumor samples into three subtypes with different survival rates and specific mutations. In summary, this study revealed 34 prognostic biomarkers and three subtypes of LIHC, which might play important roles in precision medicine regarding LIHC.

## 2. Materials and Methods

### 2.1. Datasets

**Figure 1:**
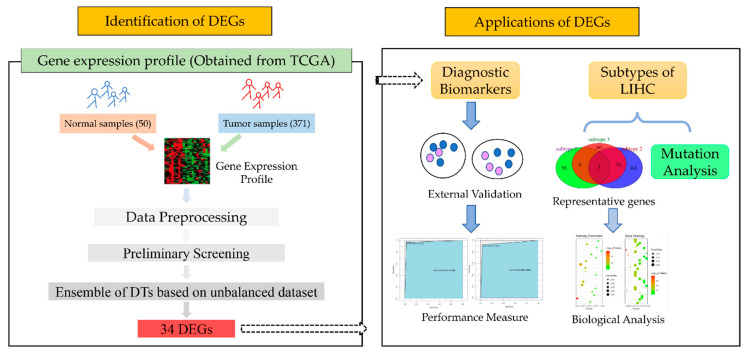
The flowchart of our main work. The process to obtain differentially expressed genes (DEGs) between tumors and normal samples is in the left panel, and the two main applications of our DEGs are in the right panel. Liver hepatocellular carcinoma (LIHC), the Cancer Genome Atlas (TCGA), and decision trees (DTs).

LIHC and HCC represent the same cancer: the most common type of primary liver cancer. The multiplatform genomics datasets including mRNA expression data, DNA-methylation data, and somatic mutation data, were downloaded from the Cancer Genome Atlas (TCGA, http://cancergenome.nih.gov/) for LIHC. The clinical information was obtained through the TCGA Data Commons (https://gdc.cancer.gov/). Two external validation datasets were downloaded from Gene Expression Omnibus (GEO, https://www.ncbi.nlm.nih.gov/gds) with accession numbers GSE39791 and GSE3500. The flowchart of the study is shown in Figure 1.

### 2.2. Data Preprocessing

As a large number of genes do not actually work in discriminating tumor samples from normal samples, we first processed the gene expression profiles based on the following three strategies: (1) We deleted genes that were unexpressed in over 50% samples including normal and tumor samples; (2) we performed maximum and minimum normalization for each gene; and (3) we replaced zero expression with a random value from the range (0, min/10), where “min” is the minimum non-zero expression value for a certain gene.

### 2.3. Identification of Differentially Expressed Genes (DEGs)

The following steps were conducted to reduce the dimension of the gene space and identify DEGs. First, we used the Fisher ratio method [17] to delete irrelevant genes. Suppose that two investigating classes (i.e., tumor and normal) on gene i have means $\mu_{i1}$, $\mu_{i2}$ and variances $\sigma_{i1}^{2}$, $\sigma_{i2}^{2}$. The Fisher ratio of gene i was defined as the ratio of the variance between the classes to the variance within the classes noted by

(1) $$Fisher\;Ratio\left(i\right)\;={\left(\mu_{i1}-\mu_{i2}\right)}^{2}/\left(\sigma_{i1}^{2}+\sigma_{i2}^{2}\right).$$

This step aimed to reject the noise genes that can only provide few information to discriminate tumors from normal samples. The Fisher ratio were estimated for each gene, then the genes with the top scores were selected.

Secondly, we calculated the spearman correlation coefficients to measure the correlation degree between genes. Genes with high correlation coefficients tended to have redundant information, which was further filtered based on the following defined classified information index [18]. The classified information index of gene i was defined as

(2) $$d(i)=\frac{1}{2}\frac{\left|\mu_{i1}-\mu_{i2}\right|}{\sigma_{i1}+\sigma_{i2}}+\frac{1}{2}\ln\left(\frac{\sigma_{i1}^{2}+\sigma_{i2}^{2}}{2\sigma_{i1}\sigma_{i2}}\right).$$

Formula (2) fully considers the influence of the mean and variance between different types of samples. Unlike Formula (1), Formula (2) could maintain genes with large differences of variances even when they have the same means in tumors and normal samples. The purpose of this step was to apply a classified informative index to screen genes with high correlation coefficients, which helped us to find the most informative genes without redundancy.

To simplify the data structure and obtain a better classification result, we applied the method of an ensemble of decision trees (DTs), which calculated feature importance using the classification performance. Due to the imbalanced dataset, this tended to select the features that had strong correlations with most classes. We used bootstrap under-sampling for LIHC to build multiple balanced datasets at the beginning. Based on each balanced dataset, we built a decision tree, and used perturbation for each gene and cross-validation to initially determine the importance score of each gene’s impact on the classification accuracy. The feature importance regarding gene j in DT i was defined by

(3) $$FI_{ij}=\frac{\sum_{k=1}^{CV}\left(ACC_{ik}-ACCF_{ijk}\right)}{CV}$$

where *CV* represents the number of cross-validations, and the accuracy before and after perturbation for gene j about the kth cross-validation in DT i are $ACC_{ik}$ and $ACCF_{ijk}$. The ensemble results of the sample classes were decided by all trees together. For example, if the results of most DTs indicated that sample j belonged to the tumor samples, then sample j was classified as a tumor sample, which was also named as a voting mechanism. The voting mechanism was also used for sample discrimination on each tree. As for the weight of the decision tree, the consistency between the prediction results of each decision tree and the integrated results of all decision trees was used as the weight of the decision tree. The weight of tree i was defined by

(4) $$TreeWeighted_{i}=\frac{\sum_{j=1}^{s}I\left(Tree_{ij}=Ensemble_{j}\right)}{S}\text{×}AccEnsemble$$

where *I* is the indicative function; $Tree_{ij}$ means the prediction result of sample j in tree i; $Ensemble_{j}$ presents the ensemble prediction result for sample j; *S* is the number of samples; and *AccEnsemble* is the accuracy of the ensemble predicted results.

Finally, a weighted combination method regarding the feature importance and the weight of DTs in each tree was proposed to finally determine the feature importance score of each gene. The feature importance score of gene j was finally defined by

(5) $$FI_{j}=\sum_{i=1}^{TN}FI_{ij}\text{×}TreeWeighted_{i}$$

where *TN* represents the number of decision trees. Then, the most important DEGs were decided based on the feature importance score as defined by Formula (5). The method described above was called the ensemble of decision trees (DTs) method.

### 2.4. Classification between Tumors and Normal Samples by DEGs

To verify whether the selected DEGs could be used as potential diagnostic biomarkers of LIHC, we first conducted hybrid sampling, which applied Synthetic Minority Oversampling TEchnique (SMOTE) over-sampling to normal samples and used k-means clustering to under-sampling the tumor samples to construct multiple balanced datasets.

Then, for each balanced dataset, we adopted a combined classifier, which was a combination of naïve bayes (NB) and support vector machine (SVM), to determine the final class of each sample by voting (Figure S1). Specifically, the TCGA dataset was divided into a training set and test set to verify our method. We also used the GSE39791 and GSE3500 datasets as independent external validation datasets. Receiver operating characteristic (ROC) curves [19] were drawn to show the performance of the classification by the selected DEGs. Hierarchical clustering was performed using the expression of DEGs in both tumors and normal samples, and the results are shown as a heat map.

### 2.5. Subtypes of LIHC

To determine the subtypes of LIHC, we performed a Bayesian clustering method with a spike-and-slab hierarchical model, which was suitable for clustering high-dimensional data using the function “bclust” in R package “e1071” [20].

The association of LIHC subtypes with the patients’ overall survival was assessed using the Kaplan–Meier survival curve and the log-rank test. To determine the representative genes of each subtype, we computed the two-side *t*-test for each gene by comparing each subtype with the other subtypes, and then selected the top 100 genes with the lowest *p*-value for each subtype. Principal component analysis (PCA) was conducted using the gene expressions of the representative genes to compare their expression profiles between the subtypes of LIHC. To further explore the biological mechanism behind subtypes, gene ontology (GO) and Kyoto Encyclopedia of Genes and Genomes (KEGG) pathway enrichment analyses were conducted for the representative genes. The gene ontology related to different subtypes in LIHC was found using the database for annotation, visualization and integrated discovery (DAVID) database (https://david.ncifcrf.gov/) for the biological process (BP), cellular component (CC), and molecular function (MF) aspects. The KEGG orthology based annotation system (KOBAS) database (http://kobas.cbi.pku.edu.cn/kobas3/genelist/) was used to detect the related KEGG pathways. Then, the enriched GO and KEGG pathways were compared between subtypes using the R package “cluster Profiler” [21].

### 2.6. Mutational Enrichment Analysis

To further investigate the mutations related to each subtype, mutational enrichment analysis was performed by pairwise and groupwise Fisher’s exact test using the function “clinical Enrichment” in R package “maftools” [22].

## 3. Results

### 3.1. Summary of Datasets

The TCGA mRNA expression dataset used in this study comprised 24,491 genes in 371 tumor samples and 50 normal samples. However, 6297 genes were deleted as they were not expressed in over 50% of the samples, following the data preprocessing mentioned in Section 2.2. Therefore, 18,694 genes were left for the study. Two external validation sets obtained from GEO were used in this study: GSE39791 comprised 72 tumor samples and 72 normal samples; and GSE3500 comprised 104 tumor samples and 76 normal samples.

There were 370 patients who had clinical information in the clinical data, and, among them, 281 patients were alive and 89 patients were dead. The information of the 370 patients was used to perform survival analysis. Only 358 patients had three types of data, including expression data, clinical information, and somatic mutations, which were used in the mutational enrichment analysis.

### 3.2. Identification of Differencially Expressed Genes (DEGs) in LIHC

The gene expression data were first normalized to identify the DEGs, after this step, as described in Section 2.3, the Fisher ratio method was performed to delete irrelevant genes. As a result, we deleted genes whose score was below 0.5, and kept 5954 genes (Figure S2).

Then, spearman correlation coefficients were calculated to measure the correlation between genes. There existed redundant genes classified by having an absolute value of the correlation coefficient greater than 0.7. To remove redundant genes, we adopted the method above, and deleted genes with a lower classified information index. After this step, we kept 1064 genes.

**Figure 2:**
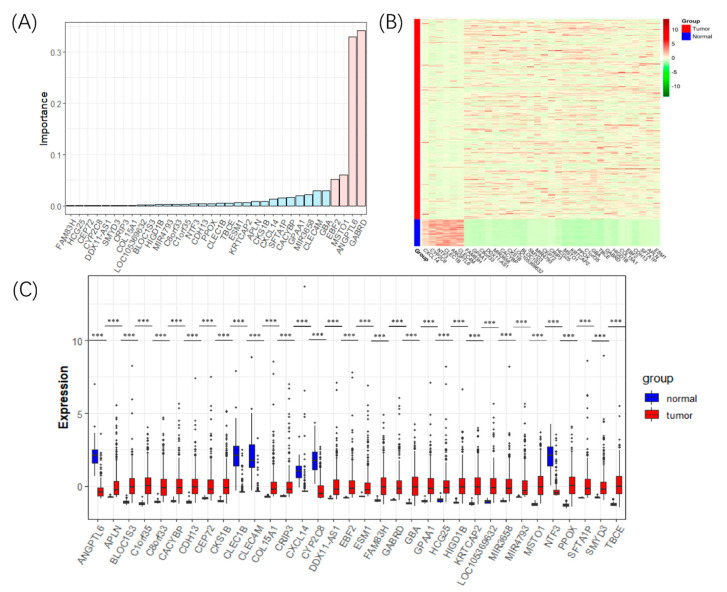
The detected 34 DEGs for LIHC. (**A**) The bar plot of the importance values of the 34 DEGs, in which the genes’ importance scores above 0.05 were marked pink, and the rest were marked sky blue. (**B**) The heat map showing the expression level of the 34 DEGs in the tumors and normal samples. (**C**) Boxplots comparing the expression level of the 34 DEGs in the tumors and normal samples. Three stars (***) marks DEGs whose *p*-value < 0.001.

Finally, 500 DTs were established using hybrid sampling. We calculated feature importance in each tree with five cross-validations and mean perturbations. Then, the importance of all genes were calculated using Formula (5), and 34 DEGs with non-zero feature importance were selected as the diagnostic biomarkers of LIHC (Figure 2A). The details of the 34 DEGs are shown in Table S1.

Twenty-eight genes of the 34 DEGs were upregulated in LIHC, including γ-aminobutyric acid type A receptor subunit delta (*GABRD*), misato mitochondrial distribution and morphology regulator 1 (*MSTO1*), EBF transcription factor 2 (*EBF2*), and Glucosylceramidase β (*GBA*), while the downregulated genes in LIHC were angiopoietin like 6 (*ANGPTL6*), C-type lectin domain family 4 member M (*CLEC4M*), C-X-C motif chemokine ligand 14 (*CXCL14*), C-type lectin domain family 1 member B (*CLEC1B*), neurotrophin 3 (*NTF3*), and cytochrome P450 family 2 subfamily C member 8 (*CYP2C8*).

We created a heat map to show the differentially expressed patterns between the tumors and normal samples of the 34 DEGs (Figure 2B). Boxplots were drawn to compare the expression of the 34 DEGs in the normal samples and LIHC samples (Figure 2C). The expression differences between the tumors and normal samples of the 34 DEGs as exhibited in both heat map and boxplots suggested that they could be used as diagnostic biomarkers for LIHC.

### 3.3. Evaluating and Comparing the Performance of Our Feature Selection Method

We randomly divided all samples into two main parts, with 80% of the samples for training and 20% of the samples for testing, and then we used K-means clustering and the SMOTE over-sampling method to constructed nine balanced datasets with a sample size of 100. Then, based on the expression profile of the 34 DEGs, we adopted a combined classifier model of NB and SVM to discriminate tumor samples from normal samples by a voting mechanism. Two external validation datasets were used to verify our method. Due to the technical differences between expression profiling by microarray (two external validation datasets) and expression profiling by high throughput sequencing (TCGA dataset), there were no expression values for some of the 34 genes in the two external validation datasets. Specifically, GSE39791 contained 29 of 34 genes, and GSE3500 contained only 23 of 34 genes.

**Figure 3:**
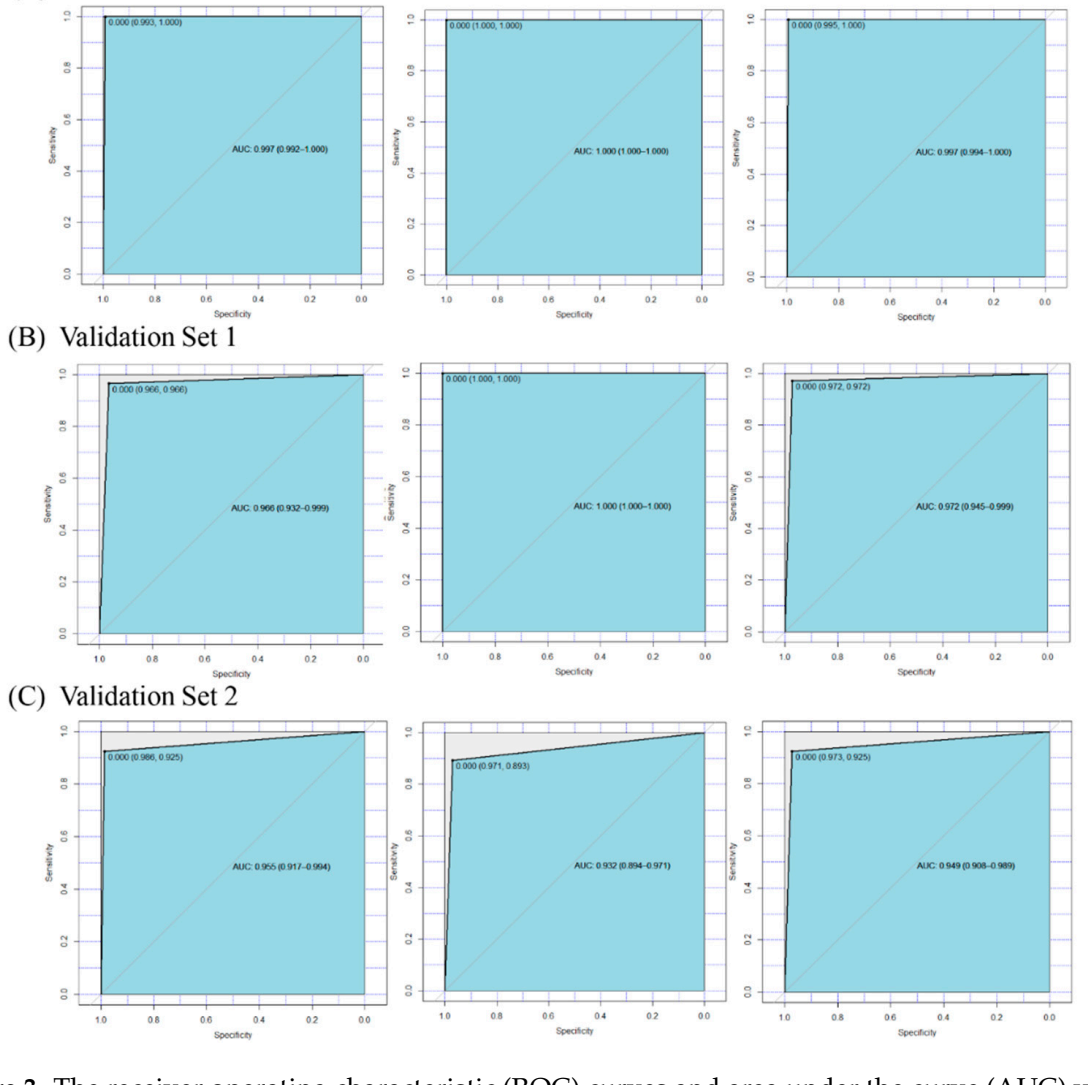
The receiver operating characteristic (ROC) curves and area under the curve (AUC) values for the TCGA dataset and two validation sets. (**A**) The results of the TCGA dataset, which contained 34 genes. (**B**) The results of validation set 1 (GSE39791), which included 29 genes. (**C**) The results of validation set 2 (GSE3500), which contained 23 genes. The left panel, middle panel, and right panel in picture (**A**–**C**) were the results of the training set, testing set, and complete set, respectively.

ROC curves were drawn to show the prediction accuracy of the DEGs to discriminate tumor samples from normal samples (Figure 3). The results showed that the 34 DEGs achieved a high area under the curve (AUC) for the training dataset (AUC = 0.997), testing dataset (AUC = 1), and complete dataset (AUC = 0.997) of the TCGA dataset (Figure 3A); twenty-nine genes in the first external validation dataset (GSE39791) also obtained a high AUC for the training dataset (AUC = 0.966), testing dataset (AUC = 1), and complete dataset (AUC = 0.972) of the GSE39791 dataset (Figure 3B); twenty-three genes in the second external validation dataset (GSE3500) also had a high AUC for the training dataset (AUC = 0.955), testing dataset (AUC = 0.932), and complete dataset (AUC = 0.949) of the GSE3500 dataset (Figure 3C). The results showed that with the number of genes increased, we could obtain a higher area under the curve (AUC) for the training, testing, and complete datasets. The accuracies of the training, testing, and complete datasets regarding TCGA data were 0.9941, 1, and 0.9952. In summary, these findings further indicated that the 34 DEGs could be potential diagnostic biomarkers of LIHC.

To show the effectiveness of our method, we compared our results with others’ (Table 1). Notably, we obtained the highest accuracy (0.9952) with 34 DEGs compared with other methods. We adopted dataset (GSE3500), which is the same dataset used in [23] to verify our method. We obtained a lower classification accuracy (0.9553) than the reference (0.9944) in the GSE3500 dataset, which may be caused by the fact that there were only 23 of 34 DEGs in the GSE3500 dataset. Therefore, 34 DEGs achieved the best performance as a whole.

### 3.4. Gene Ontology and KEGG Terms Enrichment Analysis for DEGs

**Figure 4:**
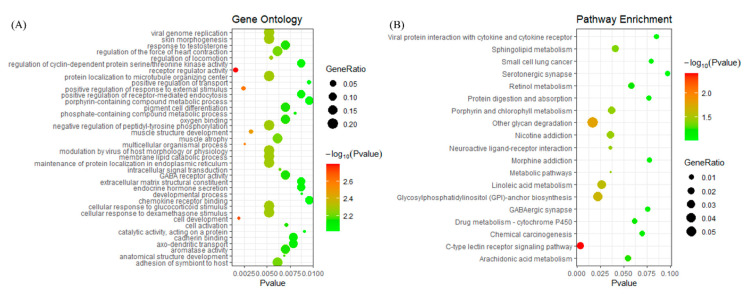
The enrichment analysis for the 34 DEGs. (**A**) The bubble diagram of the enriched gene ontology. The vertical axis represents the enrichment functions, and the horizontal axis showed the corresponding *p*-value, which is below 0.01. (**B**) The bubble diagram of the enriched Kyoto Encyclopedia of Genes and Genomes (KEGG) pathways. The vertical axis represents the enrichment pathway, and the horizontal axis shows the corresponding *p*-value, which is below 0.1.

To explore the biological mechanism behind DEGs, gene enrichment analysis was performed for the 34 DEGs. Here, we adopted gene ontology (GO) and KEGG terms to explain the mechanism of the 34 DEGs. From Figure 4A, the function annotation showed that 34 DEGs were enriched in viral genome replication, protein localization to the microtubule organizing center, the cellular response to glucocorticoid stimulus, and so on. The detailed information is shown in Supplementary Table S2. On the other hand, enriched pathways were detected for the 34 DEGs (Figure 4B). The top four enriched pathways for the 34 DEGs were the C-type lectin receptor signaling pathway, other glycan degradation, glycosylphosphatidylinositol (GPI)-anchor biosynthesis, and the linoleic acid metabolism. The genes involved in each specific pathway are shown in Supplementary Table S3. According to the results of the function and pathway enrichment analysis, the 34 DEGs were involved in important biological processes and pathways, which are related to cancer.

### 3.5. DNA Methylation Involved in Regulating the Expression of DEGs

**Figure 5:**
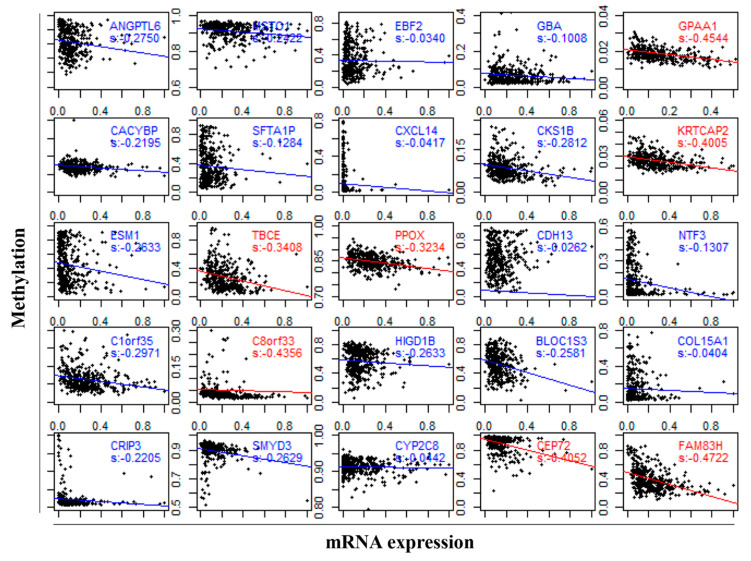
The association between the gene expression and DNA methylation of biomarkers in LIHC. The red label indicates a significant negative correlation by the Spearman test (s < −0.3), and the red line and blue line represent the regression line.

To investigate the underlying mechanisms in the regulation of the diagnostic biomarkers in LIHC, we further explored the correlation between DNA methylation and the gene expression of DEGs using the Spearman correlation coefficient. Among the 34 DEGs, only 27 genes had DNA methylation data, and 25 genes of 27 genes (92.65%) showed a negative correlation between the mRNA expression and DNA methylation (Figure 5). There was significant negative correlation between the gene expression and DNA methylation for glycosylphosphatidylinositol anchor attachment 1 (*GPAA1*, *s* = −0.4544, *p*-value = 2.2 × 10^−16^), keratinocyte associated protein 2 (*KRTCAP2*, s = −0.4005, *p*-value = 2.2 × 10^−16^), tubulin folding cofactor E (*TBCE*, s = −0.3408, *p*-value = 2.049 × 10^−11^), protoporphyrinogen oxidase (*PPOX*, s = −0.3234, *p*-value = 1.763 × 10^−10^), *C8orf33* (s = −0.4356, *p*-value = 2.2 × 10^−16^), centrosomal protein 72 (*CEP72*, s = −0.4052, *p*-value = 4.283 × 10^−16^), and family with sequence similarity 83 member H (*FAM83H*, s = −0.4722, *p*-value = 2.2 × 10^−16^), where the s indicates the value of the Spearman correlation coefficient. The results indicated that DNA methylation might contribute to the dysregulation of biomarker expression in LIHC.

### 3.6. Blust Analysis Uncovers Major Subtypes of LIHC

Applying the gene expression of the DEGs, we performed the R package “bclust” method based on the empirical Bayes method and obtained two to eight clusters. Then the k = 3 clustering solution was selected for further investigation. The k = 3 clustering solution formed three different subtypes, reference to here as “subtype 1” through “subtype 3”. The three subtypes of LIHC included: Subtype 1 with 199 cases (comparing 53.64% of tumor samples), subtype 2 with 79 cases (21.29%), and subtype 3 with 93 cases (25.07%) of LIHC cases.

**Figure 6:**
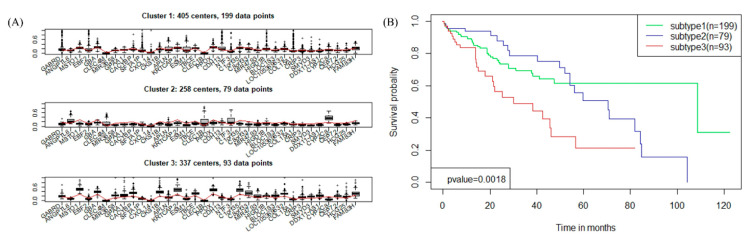
The three subtypes of LIHC. (**A**) Boxplots showing the expression level of the 34 DEGs in three subtypes. (**B**) Survival curves for three subtypes of LIHC in the TCGA dataset.

We further explored the differences in the expression patterns of 34 DEGs between the three subtypes of LIHC. The boxplots presented the expression level of 34 DEGs in different subtypes (Figure 6A), which displayed that the expression values of 34 DEGs were truly fluctuating in the three subtypes of LIHC. For example, *GABRD*, *EBF2*, Surfactant Associated 1, LncRNA (*SFTA1P*), and *GBA* had a wide range of values in subtype 1, and their changes in subtype 2 were relatively stable, while in subtype 3 they had a large range and amplitude.

Survival analysis was performed on 370 tumor samples with the clinical data (Figure 6B), which suggested that the overall survival rates in the three subtypes of patients showed significant differences (*p*-value = 1.80 × 10^−3^). The survival analysis implied that the three subtypes of LIHC could help guide clinical treatments.

### 3.7. Representative Genes of Subtypes in LIHC

**Figure 7:**
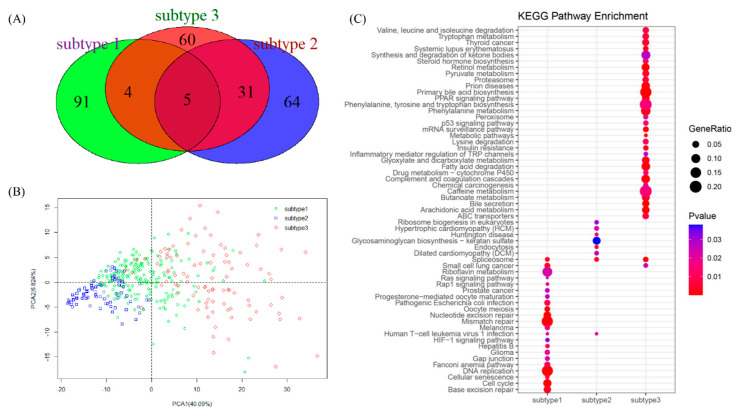
Three subtypes of LIHC. (**A**) Venn plot for the 300 representative genes of three subtypes. (**B**) Scatter plot showing the different spatial distribution of subtypes. (**C**) Comparison of the enriched KEGG pathway of representative genes for the three subtypes.

By using a two-sided *t*-test, DEGs for a given subtype versus the other two subtypes were obtained. The top 100 genes with the lowest *p*-value for each subtype were selected as the representative genes. A Venn diagram was drawn to show the distribution of the representative genes of the three subtypes (Figure 7A). As shown in Figure 7A, each subtype had many specific representative genes, even though subtype 2 and subtype 3 had 36 representative genes in common. We conducted principal component analysis (PCA) using the expression profiles of the 300 representative genes, and through the screen plot (Figure S3), we finally chose two main components and drew a scatter diagram (Figure 7B), which clearly showed that the 300 representative genes could be used to classify the three subtypes.

To reveal the pathological mechanism behind the subtypes, function enrichment analysis was performed for the representative genes of each subtype. For a detailed explanation, the enriched gene ontology (Figure S4) and KEGG pathway (Figure 7C) of the subtypes were compared, which demonstrated that the three subtypes were clearly enriched in different functions and pathways. For subtype 1, the main enriched functions included protein binding, nucleoplasm, cell division, and ATP bindings; the related KEGG pathway mainly included mismatch repair, DNA replication, cell cycle, and base excision repair. As for subtype 2, it related to the nucleus, protein binding, nucleoplasm, and so on, as well as related to the pathway of glycosaminoglycan biosynthesis-keratan sulfate. While for subtype 3, the main functions included extracellular exosomes, poly (A) RNA binding, and the nucleoplasm; it was associated with a large number of pathways, such as primary bile acid biosynthesis and phenylalanine metabolism. Therefore, different subtypes may be undergoing different tumor stages and should be treated with different methods.

### 3.8. Genetic Alteration in Subtypes

**Figure 8:**
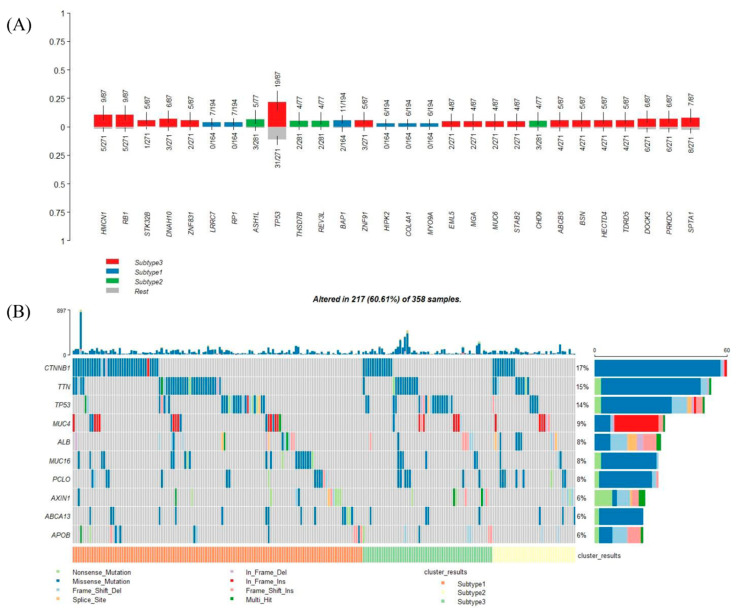
Mutation genes in different subtypes. (**A**) Mutation genes enriched in the different subtypes, ranked by the *p*-value of Fisher’s exact test. (**B**) Oncoplot showing the top 10 mutation genes in LIHC arranged by different subtypes.

Among the 358 patients with expression data, clinical information, and somatic mutations, there were 194, 77, and 87 patients for each subtype, respectively. There were 28 mutation genes enriched in different subtypes (Figure 8A). Eighteen of the 28 mutation genes (64.3%) were enriched in subtype 3, which indicated that subtype 3 tended to have more mutation genes. In this perspective, mutations could be the reason why patients in subtype 3 had the lowest survival rate. Specifically, tumor protein p53 (TP53) mutations were enriched in subtypes 3 (Figure 8A); the top 10 frequently mutated genes in LIHC were shown in Figure 8B, which also showed the detail of TP53 mutations in different subtypes.

## 4. Discussion

With the large accumulation of multi-omics data and selective molecular targeted therapies, many studies focused on the identification of biomarkers through multi-omics data analysis and provided potential biomarkers that could play important roles in the clinical management of cancer patients [29]. The discovery of biomarkers for LIHC could contribute to discriminating LIHC from normal samples. Therefore, we formed an integration method to extract the DEGs for LIHC and explored their two applications in the diagnosis and subtyping of LIHC.

We found 34 DEGs from TCGA data mainly through the ensemble of DTs, and the information regarding them was summarized in Table S1. Overall, *ANGPTL6*, *CLEC4M*, *CXCL14*, *CLEC1B*, *NTF3*, and *CYP2C8* were downregulated in LIHC, while other genes were upregulated. Some of the 34 DEGs were reported as biomarkers of HCC. For example, *CLEC4M*, *CYP2C8*, and *SFTA1P* might be valuable biomarkers for the prognosis of HCC [30,31,32]. Calcyclin Binding Protein (*CACYBP*) expression was elevated in HCC and might serve as a promising therapeutic and prognostic biomarker [33]. DDX11 Antisense RNA 1 (*DDX11-AS1*) and Apelin (*APLN*) played oncogenic role in HCC and might serve as potential therapy target for HCC [34,35]. The methylation status of the Cadherin 13 (*CDH13*) promoter in peripheral blood mononuclear cells (PBMCs) was a potential noninvasive biomarker to predict the prognosis of HCC patients [36]. Chromosome 8 Open Reading Frame 33 (*C8orf33*) was associated with the survival time of HCC patients, and could serve as a potential biomarker for distinguishing poorly differentiated from well-differentiated HCC [37]. *GABRD* could serve as a biomarker for HCC stage-IV [38]; SET And MYND Domain Containing 3 (*SMYD3*) promoted the tumorigenicity and intrahepatic metastasis of HCC cells, and could be a practical prognosis marker or therapeutic target against HCC [39]. *EBF2* might be a candidate biomarker of HCC and potential therapeutic target of it [40]. *PPOX* might act as a tumor suppressor and play a crucial role in the development of HCC [41].

Some of the 34 DEGs were related to liver function or other cancers, such as *ANGPTL6*, whose corresponding mRNA has been detected exclusively in the liver of humans [42], and a study showed that normal liver tissues produce the highest amounts of *ANGPTL6* [43]. Biogenesis of Lysosomal Organelles Complex 1 Subunit 3 (*BLOC1S3*) could induce hepatocyte apoptosis [44]. *GBA* could inhibit liver cancer, and reduce the ratio of natural killer T (NKT) lymphocytes in the liver [45]. CDC28 Protein Kinase Regulatory Subunit 1B (*CKS1B*) represented a potential research target for therapeutics of retinoblastoma [46]. *CLEC1B* and *FAM83H* were associated with a poor prognosis in LIHC [47,48]. The polymorphisms of gene *CXCL14* were linked with impaired liver function [49]. Endothelial Cell Specific Molecule 1 (*ESM1*) could serve as a biomarker for diagnosing and monitoring renal cell carcinoma [50]. *GPAA1* could be a promising diagnostic biomarker and therapeutic target for gastric cancer [51]. MicroRNA 3658 (*MIR3658*) was involved in the tumor progression of bladder cancer and had prognostic values [52]. Although *CEP72*, Chromosome 1 Open Reading Frame 35 (*C1orf35*), Collagen Type XV α 1 Chain (*COL15A1*), Cysteine Rich Protein 3 (*CRIP3*), HLA Complex Group 25 (*HCG25*), HIG1 Hypoxia Inducible Domain Family Member 1B (*HIGD1B*), *KRTCAP2*, *LOC105369632,* MicroRNA 4793 (*MIR4793*)*, MSTO1, NTF3,* and *TBCE* of the 34 DEGs have not been previously related to cancers, our study showed that they could be novel potential biomarkers of LIHC.

The pathological mechanisms of the 34 genes were mainly detected by GO and KEGG pathway analysis, and the analysis indicated that the biomarkers were enriched in metabolic-related biological processes. Altered metabolic features were found quite generally across many types of cancer cells, and a reprogrammed metabolism is considered a hallmark of cancer [53]. The cancer metabolism has been a target of cancer therapy since the appearance of chemotherapy [54]. Pathways, such as C-type lectin receptors (CLRs), are powerful pattern-recognition receptors. Additionally, a study discovered that CLRs play key roles in autoimmunity, allergies, and in maintaining homeostasis [55]. Defects in the glycosylphosphatidylinositol (GPI) biosynthesis pathway could result in a group of congenital disorders of glycosylation known as the inherited GPI deficiencies (IGDs) [56]. The pathways regarding nicotine addiction were involved in a large number of dysfunctional protein–protein interaction (PPI) pairs [57]. Neuroactive ligand–receptor interactions might play a critical role in the pathogenesis of pituitary gonadotroph adenomas [58]. The modulation of the sphingolipid metabolism and the related signaling pathways might represent a potential therapeutic approach for devastating conditions [59]. In addition, for many years, methylation was believed to play a crucial role in repressing gene expression; therefore, we further analyzed the DEG methylation in LIHC. The mRNA expression was negatively correlated with the DEG DNA methylation, which was consistent with the results found in previous studies: the presence of DNA methylation repressed gene expression in vivo [60]. Our results suggested that the expression of DEGs might be regulated by DNA methylation in LIHC.

We treated the 34 DEGs as diagnostic biomarkers of LIHC. For further analysis, we adopted two validation sets to explore the effectiveness of the 34 biomarkers. Though the two datasets contained only a subsect of the 34 DEGs, we discovered possible tendencies for the 34 DEGs: When the number of DEGs increased, we obtained higher AUC values in the training set, testing set, and complete set, which suggested that the 34 DEGs could be potential biomarkers of LIHC.

We applied the 34 DEGs to identify subtypes of LIHC, and discovered three subtypes of LIHC that were significantly associated with overall survival (*p*-value = 1.8 × 10^−3^). Based on this step, we detected the representative genes of each subtype, and applied function and KEGG pathway enrichment analysis. For Subtype 1, pathways were mainly associated with the cell cycle, DNA replication, and mismatch repair, which implied that subtype 1 had disorder in the cell cycle processes. For subtype 2, pathways, such as endocytosis and the spliceosome, were enriched, and research reported that the endocytosis and spliceosome pathways could represent the first signs of embryonic activity. With the help of the spliceosome, endocytosis acted as one of main activators for finding a series of successive waves of maternal pioneer signal regulators [61]. The misregulation of endocytosis could result in HCC [62].

As for subtype 3, the enriched pathways played important roles in liver cancer; for example, metabolic pathways that were reported as therapeutic targets in liver cancer [63]; complement and coagulation cascades, which were crucially involved in the inflammatory response [64]; and the retinol metabolism, which plays important roles in the development of the nervous system, notochord, and other embryonic structures, and in the maintenance of epithelial surfaces, immune competence, and reproduction [65]. In summary, the above analysis suggested that the three subtypes of LIHC displayed different biological processes.

We divided the 371 tumor samples into three subtypes according to the gene expression profiles of the identified 34 DEGs. Although these 371 samples contained three subtypes of histology including HCC, fibrolamellar carcinoma, and mixed hepatocellular cholangiocarcinoma, almost 97.3% of them were HCC. Explaining the detailed relationship between our defined molecular subtypes and the histology subtypes is, thus, difficult. In addition, we drew a heat map for the molecular subtypes, pathologic stage, and the TNM classification of malignant tumors (TNM) staging in Figure S5. Specifically, the percentages of pathologic stage I and II are 71.9% (143/199) in subtype 1, 69.6% (55/79) in subtype 2, and 61.3% (57/93) in subtype 3, respectively; the percentages of pathologic stage III and IV are 20.6% (41/199) in subtype 1, 22.8% (18/79) in subtype 2, and 33.3% (31/93) in subtype 3, respectively. Although, there was no obvious evidence to show inevitable correlation between molecular subtypes and pathologic stages, the data suggested that subtype 1 had a higher proportion of pathologic stage I and II, while subtype 3 had a higher proportion of pathologic stage III and IV, which could help us understanding the three molecular subtypes better. However, we just briefly analysed the relationship between molecular subtypes and pathologic stage at the data level, there might have some inner connection we didn’t find by our method. Therefore, further study about it is necessary. From future perspective, the molecular subtypes of LIHC will change the traditional classification methods and treatment strategies. With the development of data science and modern medicine, the molecular subtypes will be better defined and hopefully be applied to clinic.

In summary, our research findings revealed the diagnostic biomarkers of LIHC, and determined the subtypes. Considering future research, we must admit the limitations of this paper: first, the thresholds in the feature selection process were set from experience; second, the external validation sets in this study contained only a part of the DEGs; however, no dataset containing all 34 DEGs was found.

## 5. Conclusions

In this study, we proposed a new framework and identified 34 DEGs that could discriminate tumor samples from normal samples, and, through validation and enrichment analysis, we verified that some of the 34 DEGs could serve as novel diagnostic biomarkers for LIHC. We further analyzed the 34 DEGs, and applied them to divide LIHC into three subtypes that might contribute to the accuracy judgement during the treatment of LIHC. The identified representative genes of each subtype could be potentially targetable markers for the different subtypes. In addition, mutations associated with the subtypes could be potential markers for drug development. Therefore, our results could aid in realizing future personalized medicine for LIHC.

## Figures and Tables

**Table 1:** Comparison of classification results obtained by different methods in the hepatocellular carcinoma (HCC) dataset.

No.	Methods	Validation Methods	No. of DEGs	Accuracy	Source
1	Ensemble of DT based on unbalanced dataset	Naïve Bayes (NB) and support vector machine (SVM)	34	0.9952	Our method
2	Significance Analysis of Microarrays (SAM)-*t* test + Gene regulatory probability (GRP)	SVM (RBF, Radial Basis Function Kernel)	10	0.9944	[23]
3	Semi-supervised gene selection	Spectral Biclustering	1	0.9870	[24]
4	*t*-test + class separability	Fuzzy neural network	2	0.9810	[25]
5	Univariate cox regression and Lasso penalized cox regression analysis	Kaplan–Meier	12	0.9311	[26]
6	Resampling + SAM	K nearest neighbor	10	0.93	[27]
7	Meta Threshold Gradient Descent Regularization	Logistic regression	34	0.8400	[28]

## Supplementary Materials

The following are available online at https://www.mdpi.com/2073-4425/11/9/1051/s1, Figure S1: The flowchart regarding the classification of the samples; Figure S2: The results of the Fisher ratio; Figure S3: Screen plot of 300 representative genes; Figure S4: The bubble diagram of functional enrichment; Figure S5: The heatplot for the molecular subtypes, pathologic stage, and TNM staging; Table S1: The detailed information for the 34 DEGs; Table S2: Function enrichment analysis of the 34 DEGs; Table S3: Pathway enrichment analysis of the 34 DEGs.
