# Supplementary material for: Identification of Diagnostic Biomarkers and Subtypes of Liver Hepatocellular Carcinoma by Multi-Omics Data Analysis
Source: Genes (Basel). 2020 Sep 6;11(9):1051. doi: 10.3390/genes11091051 (PMC7566011; doi:10.3390/genes11091051)
Supplement: Supplementary file 1 [file genes-11-01051-s001.pdf]

Supplementary Figures and Tables

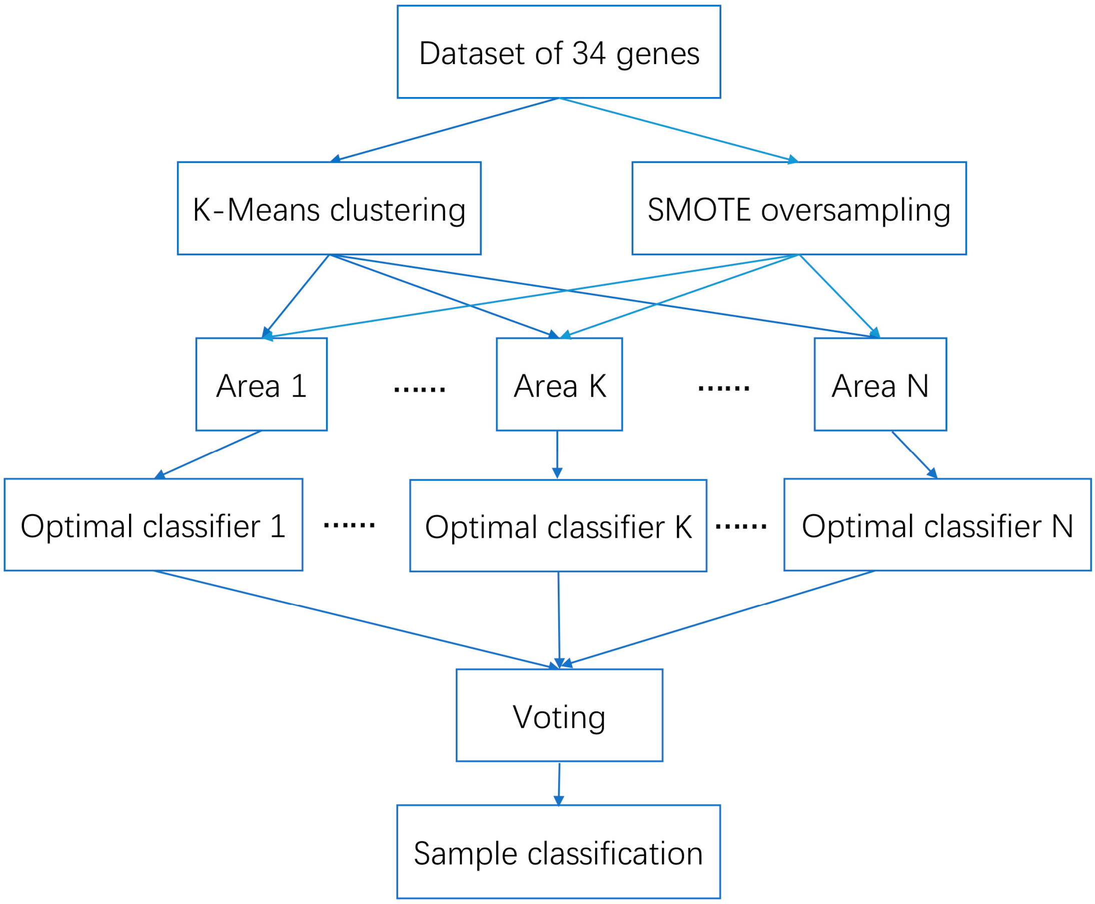

Figure S1. The flowchart regarding the classification of the samples. The detailed operating procedures and information about classification of samples.

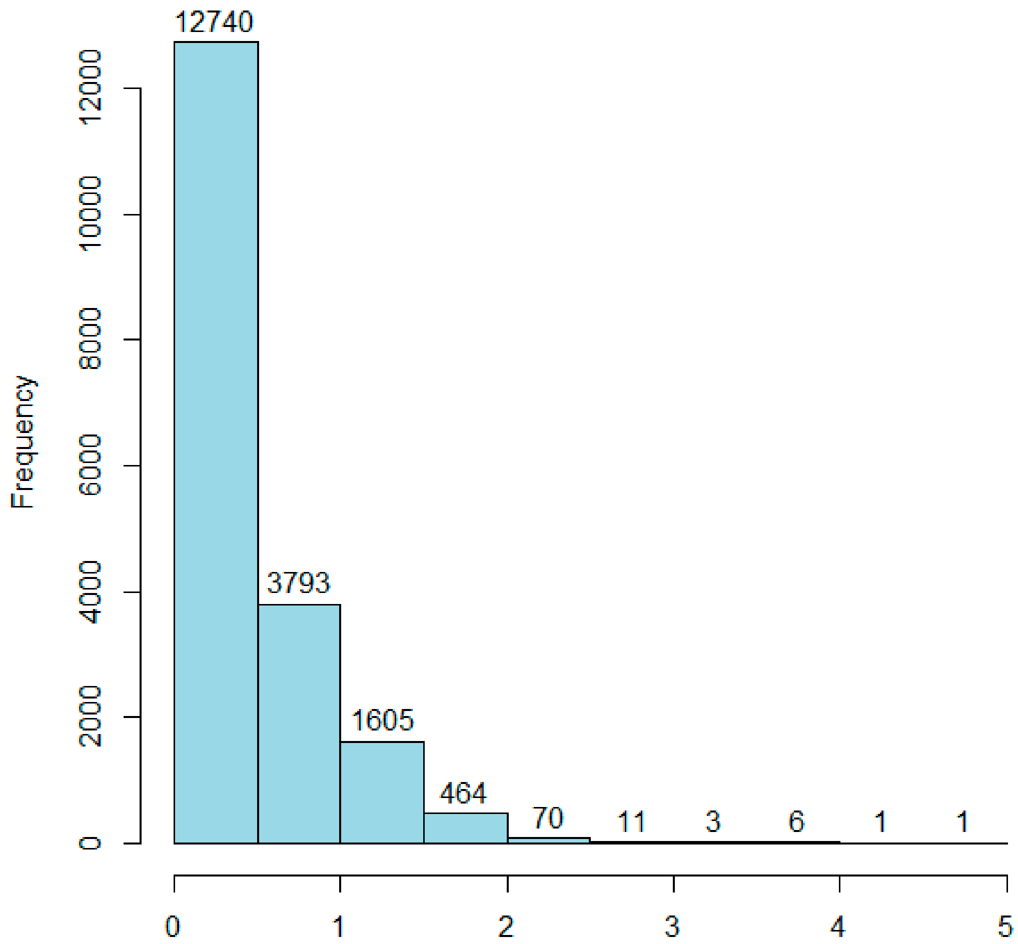

Figure S2. The results of the Fisher Ratio. Bar plot presented the interval of genes' fisher ratio and the number of genes in each interval.

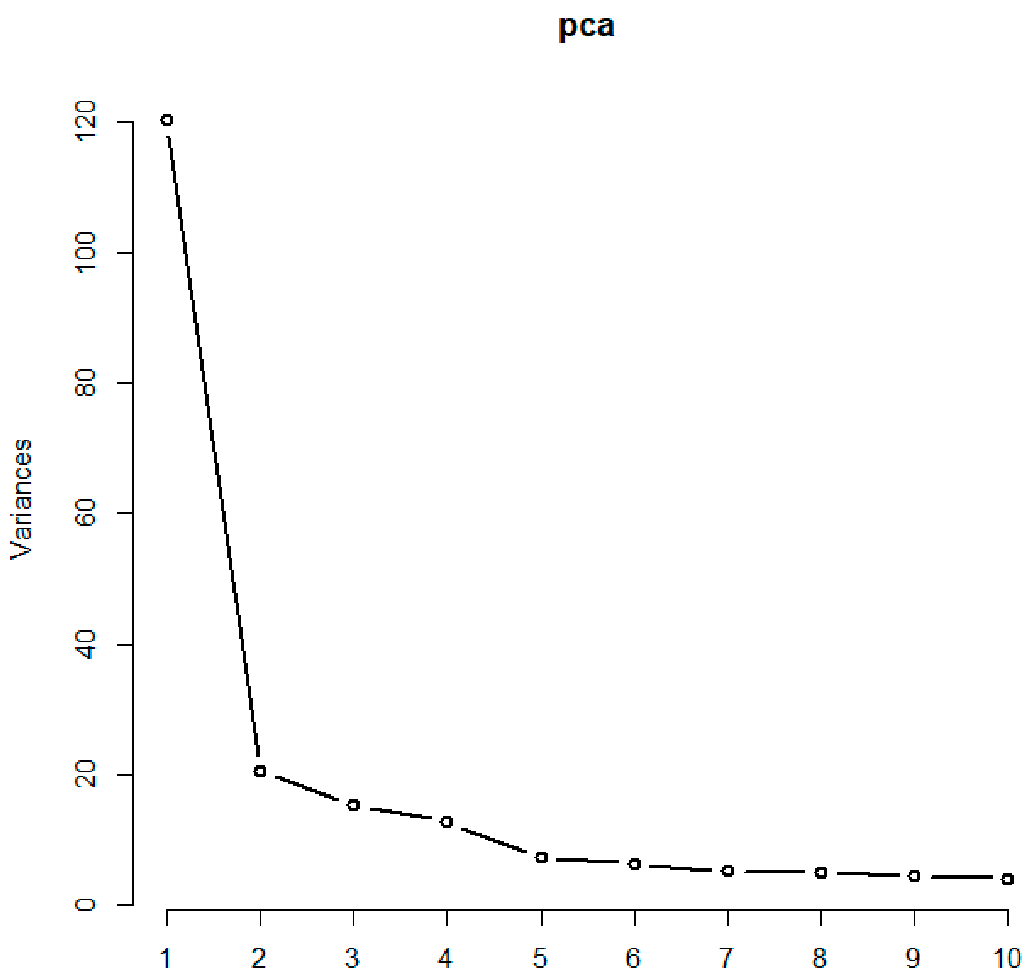

Figure S3. Screen plot of 300 representative genes. Scree plot was drawn to show the correlation between variance and the number of main components.

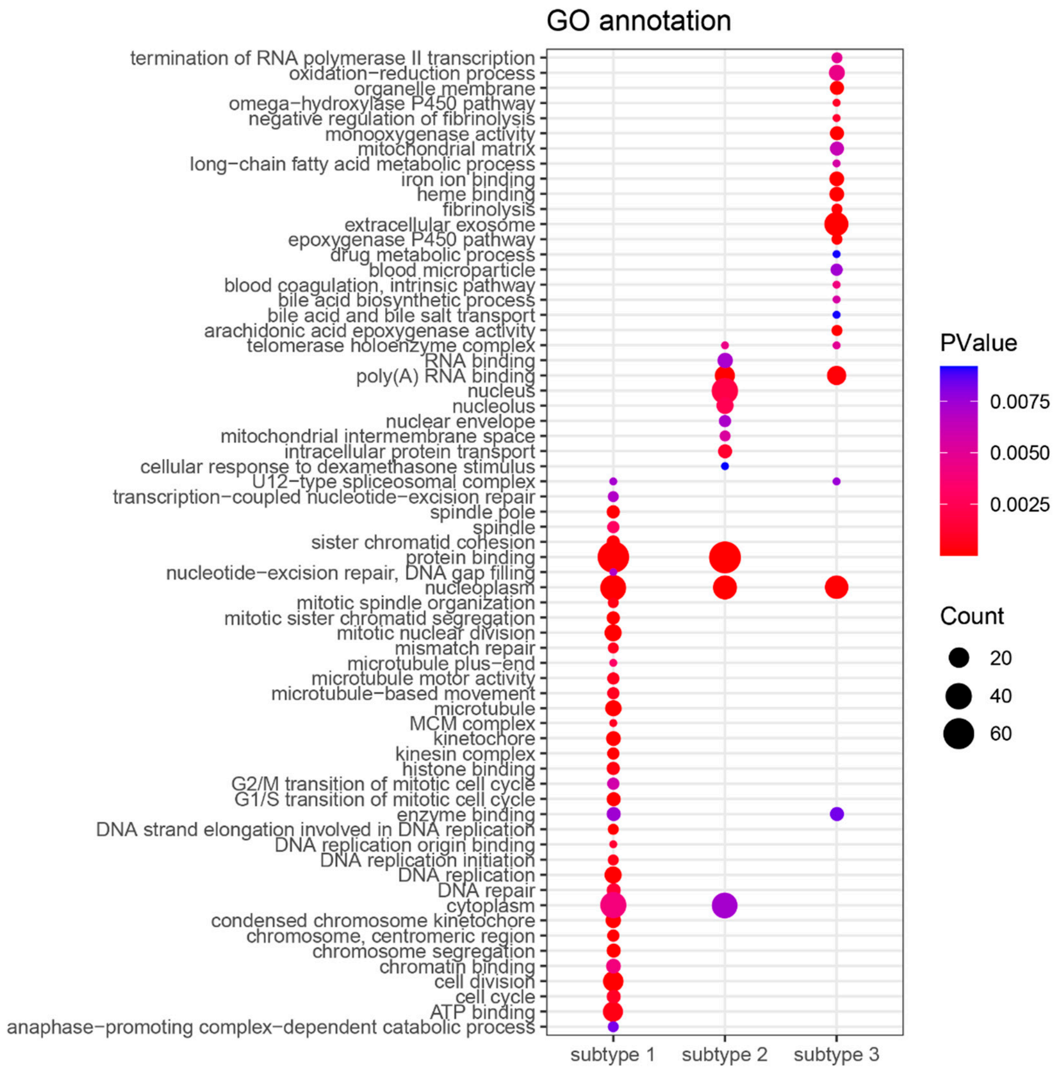

Figure S4. The bubble diagram of functional enrichment. The enriched Gene Ontology for subtype 1, subtype 2 and subtype 3. Count means the number of genes enriched in a certain annotation.

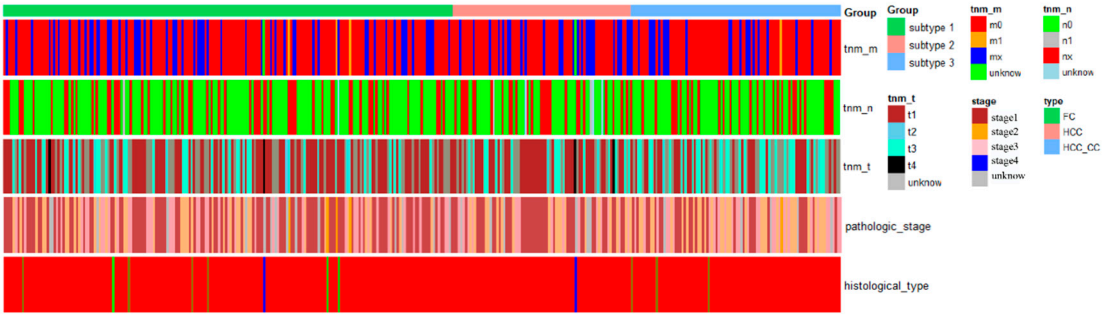

Figure S5. The heatplot for the molecular subtypes, pathologic stage and TNM staging. Tnm_m, tnm_n and tnm_t were the TNM staging.

Table S1. The detailed information for the 34 DEGs.

Gene Symbol	Discription	Reference	*p*-Value	Fold Change
GABRD	stage-specific differentially expressed in hepatocellular carcinoma	[38]	3.368509×10^{-62}	34.8256
ANGPTL6	highly expressed in liver cancer	[43]	1.338193×10^{-23}	0.1879
MSTO1	Required for mitochondrial fusion and mitochondrial network formation	GeneCards	2.034419×10^{-94}	5.1055
EBF2	A potential therapeutic target of HCC	[40]	3.463284×10^{-49}	56.8254
GBA	inhibit liver cancer	[45]	9.070282×10^{-83}	4.0856
CLEC4M	a valuable biomarker for the prognosis of the patients with HCC	[30]	2.854162×10^{-14}	0.0273
MIR3658	involved in tumor progression of bladder cancer	[52]	4.156083×10^{-68}	5.0915
GPAA1	a therapeutic target for gastric cancer	[51]	1.874503×10^{-70}	4.0789
CACYBP	highly expressed and associated with poor prognosis in HCC	[33]	6.909608×10^{-69}	3.3634
SFTA1P	significant association with overall survival in HCC patients	[32]	3.449638×10^{-48}	123.5817
CXCL14	linked with impaired liver function	[49]	5.819188×10^{-20}	0.1507
CKS1B	represented a potential research target for therapeutics of retinoblastoma	[46]	9.562192×10^{-68}	3.7726
APLN	played an oncogenic role in HCC	[35]	1.226416×10^{-42}	24.4214
KRTCAP2	The gene is overexpressed in Liver	GeneCards	8.404593×10^{-88}	3.4930
ESM1	serve as a biomarker for diagnosing and monitoring renal cell carcinoma	[50]	1.897959×10^{-37}	29.5066
TBCE	Low tissue specificity	The Human Protein Atlas	2.562632×10^{-92}	3.3832
CLEC1B	a signature gene highly associated with tumor progression	[47]	9.306855×10^{-17}	0.0368
PPOX	played a crucial role in the development of HCC	[41]	8.340677×10^{-97}	3.5038
CDH13	a potential noninvasive biomarker of HCC	[36]	7.580294×10^{-71}	7.8091
NTF3	controls survival and differentiation of mammalian neurons	GeneCards	1.135516×10^{-22}	0.1380

C1orf35	related to pathway Innate Immune System	GeneCards	9.410057 × 10^{-86}	3.9379
C8orf33	correlated with overall survival in HCC patients	[37]	5.074940 × 10^{-74}	3.7787
MIR4793	involved in post-transcriptional regulation of gene expression in multicellular organisms	GeneCards	2.573851 × 10^{-44}	17.7375
HIGD1B	links to tumorigenesis and the progression of pituitary adenomas	GeneCards	1.753135 × 10^{-79}	13.1410
BLOC1S3	induce hepatocyte apoptosis	[44]	1.733118 × 10^{-74}	3.1598
LOC105369632	an RNA Gene, affiliated with the lncRNA class	GeneCards	1.855580 × 10^{-69}	4.5705
COL15A1	Prognostic marker in liver cancer (favourable)	The Human Protein Atlas	9.020337 × 10^{-41}	21.5720
CRIP3	Cancer enhanced (liver cancer)	The Human Protein Atlas	2.201642 × 10^{-35}	7.0812
SMYD3	promoted the tumor igenicity and intrahepatic metastasis of HCC cell	[39]	3.849815 × 10^{-46}	4.5849
DDX11-AS1	played important role during HCC oncogenesis	[34]	7.968983 × 10^{-52}	12.1320
CYP2C8	downregulated in HCC and could be a potential prognostic biomarker	[31]	2.130738 × 10^{-20}	0.2245
CEP72	the major microtubule-organizing center in animal cells	GeneCards	6.352548 × 10^{-48}	4.2004
HCG25	an RNA Gene, and is affiliated with the lncRNA class	GeneCards	5.731509 × 10^{-59}	5.1442
FAM83H	expressed higher in HCC cells compared to normal liver	[48]	1.191638 × 10^{-63}	4.2419

Table S2. Function enrichment analysis of the 34 DEGs.

**Function**	***p-Value***	**Genes**
Receptor regulator activity	1.592 × 10^{-3}	*NTF3,CXCL14*
Cell development	1.927 × 10^{-3}	*NTF3, TBCE, SMYD3*
Positive regulation of response to external stimulus	2.429 × 10^{-3}	*NTF3, CDH13*
Multicellular organismal process	2.607 × 10^{-3}	*NTF3,GBA,ANGPTL6, TBCE, CDH13*
Muscle structure development	3.298 × 10^{-3}	*CXCL14, SMYD3*
Viral genome replication	5.250 × 10^{-3}	*CLEC4M*
Membrane lipid catabolic process	5.250 × 10^{-3}	*GBA*
Cellular response to glucocorticoid stimulus	5.250 × 10^{-3}	*SMYD3*
Modulation by virus of host morphology or physiology	5.250 × 10^{-3}	*CLEC4M*
Protein localization to microtubule organizing center	5.250 × 10^{-3}	*CEP72*

Cellular response to dexamethasone stimulus	5.250×10^{-3}	*SMYD3*
Skin morphogenesis	5.250×10^{-3}	*GBA*
Maintenance of protein localization in endoplasmic reticulum	5.250×10^{-3}	*GPAA1*
Negative regulation of peptidyl-tyrosine phosphorylation	5.250×10^{-3}	*NTF3*
Regulation of locomotion	5.405×10^{-3}	*NTF3, CDH13*
regulation of the force of heart contraction	6.122×10^{-3}	*APLN*
Adhesion of symbiont to host	6.122×10^{-3}	*CLEC4M*
Muscle atrophy	6.122×10^{-3}	*TBCE*
Intracellular signal transduction	6.400×10^{-3}	*CLEC4M,NTF3, GBA*
Anatomical structure development	6.891×10^{-3}	*NTF3,COL15A1,CLEC1B, CXCL14*
Pigment cell differentiation	6.993×10^{-3}	*BLOC1S3*
Aromatase activity	6.993×10^{-3}	*CYP2C8*
Oxygen binding	6.993×10^{-3}	*CYP2C8*
GABA receptor activity	6.993×10^{-3}	*GABRD*
Response to testosterone	6.993×10^{-3}	*GBA*
Cell activation	7.107×10^{-3}	*CLEC1B, BLOC1S3*
Cadherin binding	7.864×10^{-3}	*CDH13*
Axo-dendritic transport	7.864×10^{-3}	*BLOC1S3*
Phosphate-containing compound metabolic process	8.058×10^{-3}	*NTF3,GBA,GPAA1*
Regulation of cyclin-dependent protein serine/threonine kinase activity	8.734×10^{-3}	*CKS1B*
Positive regulation of receptor-mediated endocytosis	8.734×10^{-3}	*NTF3*
Extracellular matrix structural constituent	8.734×10^{-3}	*COL15A1*
Endocrine hormone secretion	8.734×10^{-3}	*APLN*
Developmental process	8.799×10^{-3}	*FAM83H,CLEC1B, EBF2,BLOC1S3*
Catalytic activity, acting on a protein	9.024×10^{-3}	*SMYD3,GPAA1,CKS1B*
Positive regulation of transport	9.557×10^{-3}	*NTF3, APLN*
Chemokine receptor binding	9.604×10^{-3}	*CXCL14*
Porphyrin-containing compound metabolic process	9.604×10^{-3}	*PPOX*

Table S3. Pathway enrichment analysis of the 34 DEGs.

**Pathway**	***P-Value***	**Genes**
C-type lectin receptor signaling pathway	3.9220×10^{-3}	*CLEC4M, CLEC1B*
Other glycan degradation	1.6532×10^{-2}	*GBA*
Glycosylphosphatidylinositol (GPI)-anchor biosynthesis	2.2556×10^{-2}	*GPAA1*
Linoleic acid metabolism	2.5981×10^{-2}	*CYP2C8*
Nicotine addiction	3.5343×10^{-2}	*GABRD*
Neuroactive ligand-receptor interaction	3.5760×10^{-2}	*GABRD, APLN*
Metabolic pathways	3.6029×10^{-2}	*CYP2C8,GBA, PPOX, GPAA1*
Porphyrin and chlorophyll metabolism	3.7036×10^{-2}	*PPOX*

Sphingolipid metabolism	4.1255×10^{-2}	*GBA*
Arachidonic acid metabolism	5.4635×10^{-2}	*CYP2C8*
Retinol metabolism	5.7952×10^{-2}	*CYP2C8*
Drug metabolism - cytochrome P450	6.2082×10^{-2}	*CYP2C8*
Chemical carcinogenesis	7.0290×10^{-2}	*CYP2C8*
GABAergic synapse	7.5993×10^{-2}	*GABRD*
Protein digestion and absorption	7.6805×10^{-2}	*COL15A1*
Morphine addiction	7.7617×10^{-2}	*GABRD*
Small cell lung cancer	7.9237×10^{-2}	*CKS1B*
Viral protein interaction with cytokine and cytokine receptor	8.4888×10^{-2}	*CXCL14*
Serotonergic synapse	9.6883×10^{-2}	*CYP2C8*
